# Macrophages regulate healing-associated fibroblasts in diabetic wound

**DOI:** 10.1007/s11033-023-09100-1

**Published:** 2024-01-25

**Authors:** Yu Xiao, Jieqi Qian, Xiaohui Deng, Huifeng Zhang, Jiancheng Wang, Zhijun Luo, Lingyan Zhu

**Affiliations:** 1 Department of Endocrinology and Metabolism, The First Affiliated Hospital of Nanchang University, 17 Yongwaizheng Street, Nanchang, 330006 China; 2 North Allegheny High School, Wexford, PA 15090 USA; 3 Department of Ultrasound in Medicine, The Second Affiliated Hospital Zhejiang University School of Medicine, Zhejiang University, Hangzhou, 310009 China; 4 Jiangxi Clinical Research Center for Endocrine and Metabolic Disease, The First Affiliated Hospital of Nanchang University, Nanchang, 330006 China; 5 Department of Endocrinology, The Henan Provincial People’s Hospital, Zhengzhou, 450003 China; 6 Jiangxi Province Key Laboratory of Tumor Pathogenesis and Molecular Pathology, Nanchang University, Nanchang, 330031 China; 7 Queen Mary School, Nanchang University, Nanchang, 330031 China; 8 Academic Affairs Office of the First Affiliated Hospital of Nanchang University, Nanchang, China

**Keywords:** Diabetic foot ulcers (DFUs), Diabetic wound (DW), Macrophages, Fibroblasts, Interleukin-6 (IL6), Macrophage polarization

## Abstract

**Background:**

Recovery from a foot ulcer is compromised in a diabetic status, due to the impaired tissue microenvironment that consists of altered inflammation, angiogenesis and fibrosis. Phenotypic alterations in both macrophages and fibroblasts have been detected in the diabetic wound. Recently, a fibroblast subpopulation that expresses high matrix metalloproteinase 1 (MMP1), MMP3, MMP11 and Chitinase-3-Like Protein 1 (CHI3L1) was associated with a successful diabetic wound healing. However, it is not known whether these healing-associated fibroblasts are regulated by macrophages.

**Methods and Results:**

We used bioinformatic tools to analyze selected public databases on normal and diabetic skin from patients, and identified genes significantly altered in diabetes. In a mouse model for diabetic wound healing, we detected not only a loss of the spatiotemporal changes in interleukin 1β (IL1β), IL6, IL10 and vascular endothelial growth factor A (VEGF-A) in wound macrophages, but also a compromised expression of MMP1, MMP3, MMP11, CHI3L1 and VEGF-A in healing-associated wound fibroblasts in a diabetic status. Co-culture with diabetic macrophages significantly reduced the expression of MMP1, MMP3, MMP11, CHI3L1 and VEGF-A in fibroblasts from non-diabetic wound. Co-culture with non-diabetic macrophages or diabetic macrophages supplied with IL6 significantly increased the expression of MMP1, MMP3, MMP11, CHI3L1 and VEGF-A in fibroblasts from diabetic wound. Moreover, macrophage-specific expression of IL6 significantly improved wound healing and angiogenesis in diabetic mice.

**Conclusions:**

Macrophages may induce the activation of wound-healing-associated fibroblasts, while the defective macrophages in diabetes may be corrected with IL6 treatment as a promising therapy for diabetic foot disease.

## Introduction

More than 300 million people suffer from severely defective glucose metabolism, a disease called diabetes [1]. A long-term hyperglycemic status causes severe complications in diabetic patients [2]. Non-healing wounds, which typically occur in the feet of the patients known as diabetic foot ulcers (DFUs), are the most severe diabetic complication and a leading cause of amputations, resulting in more than a 50% mortality in five years [3]. Thus, treatment of DFUs constitutes the highest annual US medical expense [4].

Wound healing is a complicated process encompassing a hemostasis phase, inflammation phase, proliferation phase and remodeling phase in a linear, coordinated and partially overlapping manner. Wound healing is orchestrated by cooperations from many different cell types, including macrophages and fibroblasts [5]. Macrophages, key immune cells, arrive first at the injury site, clearing debris and pathogens. Their secreted cytokines and growth factors then recruit and activate fibroblasts, essential for tissue repair and scar formation. Fibroblasts synthesize extracellular matrix components, crucial for structural support and wound closure. Macrophages undergo a transformation from a pro-inflammatory (M1) to a healing-promoting (M2) phenotype, guided by the wound environment [5]. M2 macrophages release anti-inflammatory cytokines and growth factors that further stimulate fibroblasts for collagen deposition, angiogenesis, and wound contraction. Defective or dysfunctional macrophages are believed to contribute significantly to the impaired wound healing in diabetes [6]. In the inflammatory phase of wound healing, macrophages receive local signals to be polarized into a proinflammatory/M1 type, during which they produce and secrete cytokines such as IL1β, IL6, IL10, IL12, IFNɣ and TNFα [5] to promote the progression of inflammation and clearance of dead tissue and cell debris. In the proliferative phase of the wound healing, a dynamic change of macrophage phenotype results in the domination of anti-inflammatory/M2 macrophages in the wound and their promotion of cell proliferation, wound repair and tissue remodeling [6]. The abnormal macrophage phenotype in diabetes is believed to mainly result from the epigenetic changes in macrophages and their impaired crosstalk with other cell types, such as neutrophils, fibroblasts, endothelial cells and lymphocytes [7–13].

Interleukin-6 (IL-6) plays a significant role in this context. Initially, it acts as a pro-inflammatory cytokine, crucial in the early phase of wound healing [14]. It promotes the recruitment of immune cells, including macrophages, to the injury site [14]. However, IL-6 also aids in the transition of macrophages from an M1 to an M2 phenotype [15]. This shift is vital for downregulating inflammation and facilitating the healing process [15]. Furthermore, IL-6 directly influences fibroblast activity, enhancing their proliferation and migration, which are vital for tissue repair [16]. Thus, IL-6 presents as a therapeutic agent that can potentially modulate both macrophage function and fibroblast activity, addressing different stages of the wound healing process, thereby accelerating tissue repair and regeneration [16].

Very recently, a single cell transcriptomic study has revealed a unique fibroblast subpopulation that expresses high matrix metalloproteinase 1 (MMP1), MMP3, MMP11, Chitinase-3-Like Protein 1 (CHI3L1) in successful diabetic wound healing associated with greater M1 macrophage polarization [17]. MMPs are enzymes that play a key role in extracellular matrix (ECM) remodeling, crucial for wound closure and tissue repair. They help in breaking down damaged ECM components, enabling cellular migration and new tissue formation [18]. However, in diabetes, MMP activity can be dysregulated, leading to impaired wound healing [19]. CHI3L1 is another significant molecule involved in tissue remodeling and inflammation. It is known to modulate cellular responses during tissue repair, including fibroblast proliferation and angiogenesis [19]. However, a possible regulation of the healing-associated fibroblasts by macrophages has not been investigated and is thus addressed in this study.

## Materials and methods

### Protocol approval

All the protocols including in vitro cell studies and in vivo animal work have been approved by the institutional research committee of Nanchang University. Inbred littermate mice were used to minimize experimental confounder, while the number of mice used in each experiment was determined by a power calculation (p < 0.05).

### Production of adeno-associated viruses (AAVs)

Plasmids for generating AAVs in this study were obtained from Addgene (Watertown, MA, USA). They were a backbone plasmid (#32395 [20]), a CD68 promoter plasmid (#34837 [21]), a mouse IL6 plasmid (IL6-GFP, #28088 [22]). The sequence of a control scramble construct was 5’-GCCCTTATTAACGGTATTAACGCCGGCTTUAAGCC-3’. Serotype 6 was chosen to carry the transgenes since we have shown that it was very efficient for transducing macrophages [23]. Transfection of human embryonic kidney 293 cells with the prepared plasmids, purification of AAVs and titration of AAVs by a dot-blot assay were done as described before [23].

### Induction of diabetes, DFD and AAV transplantation

Diabetes was induced in 12-week-old C57/Bl6 mice (SLAC Laboratory Animal, Shanghai, China) by single intraperitoneal (i.p) injection of 150 mg/kg streptozotocin (STZ) in 120µl normal saline after an overnight fasting, as described before, which was proven to induce hyperglycemia in mice (fasting blood glucose >  = 350 mg/dl) [23]. Equal volume of the normal saline was i.p. given to some mice as a control for STZ. One week later, a round wound (2cm in diameter) was created on the dorsal midline of the STZ-treated diabetic mice, which received orthotopic injection with 80µl saline as controls or AAVs [2×10^11^ genome copy particle (GCP)/ml], as described [23]. Four groups of mice of 5 each were applied in the experiment. Group 1: mice received i.p. saline and local saline (saline + saline). Group 2: mice received i.p. STZ and local saline (STZ + saline). Group 3: mice received i.p. STZ and local control virus (STZ + AAV-pCD68-Scr); Group 4: mice received i.p. STZ and local IL6 virus (STZ + AAV-pCD68-IL6). Blood glucose level was measured after an overnight fasting. The pancreatic beta-cell mass was calculated as the product of the pancreas weight and the % insulin-positive area as described [23]. For labeling functional vessels, tail vein injection of a tomato-lectin (50 µl, Vectorlabs, Burlingame, CA USA) was performed 8 min before sacrifice of the mice. Vessel density was assessed and presented as the ratio of lection-positive area to the total tissue area, as described before [23].

### Flow cytometry

The wound tissue was dissected out and digested with 0.3% trypsin for 25–30 min to get completely dissociated for flow cytometry. Cell sorting was based on immunofluorescence for F4/80 (by a PE-conjugated anti-F4/80 antibody, #565,410, Becton–Dickinson Biosciences, San Jose, CA, USA) or DLK1 (unconjugated, ab119930, Abcam, Cambridge, MA, USA; followed by incubation with an anti-mouse BV421-conjugated 2nd antibody) or CD163 (by an APC-conjugated anti-CD163 antibody, #17-1631-82, eBioscience, San Diego, CA, USA). A Flowjo software (version 12, Flowjo LLC, Ashland, OR, USA) was used for analyzing and presenting data.

### Co-culture

Macrophages and fibroblasts isolated from the wound tissue were co-cultured using a transwell system featuring a 0.4 µm pore size insert membrane (ThermoFisher Scientific, Pittsburgh, PA, USA). This setup allowed for the exchange of growth factors and cytokines but prevented the physical intermingling of the cells. To initiate the co-culture, Macrophages were seeded in the lower chamber, while fibroblasts were placed in the upper insert. The cells were cultured in their respective media at 37 °C with 5% CO2. IL-6, obtained from R&D Biosystems (Shanghai, China), was added to the co-culture at a concentration of 0.1 ng/ml. This concentration was chosen based on literature and preliminary dose–response experiments to optimize cellular response without inducing cytotoxicity. The co-culture was maintained for 48 h, to allow sufficient time for cell–cell signaling via the secreted factors. Post-culture, the cells were harvested separately from each compartment for subsequent analyses.

### ELISA

The total protein was extracted from tissue or cells using a CelLytic™ MEM Protein Extraction Kit (CE0050, Sigma-Aldrich, St. Louis, MO, USA). Enzyme-linked immunosorbent assays (ELISAs) were done for IL1β (ab197742; Abcam), tumor necrosis factor alpha (TNFα, ab208348; Abcam), interferon gamma (IFNɣ, ab282874; Abcam), IL6 (Ab100713; Abcam), IL10 (M1000B; R&D Biosystems), CD163 (ab272204; Abcam), transforming growth factor β1 (TGFβ1, ab119557; Abcam), VEGF-A (ab209882; Abcam), fibroblast growth factor 1 (FGF1, ab223587; Abcam), MMP1 (NBP3-06885; Novus Biologicals, Centennial, CO, USA), MMP3 (ab203364; Abcam), MMP11 (NBP3-06935; Novus Biologicals) and CHI3L1 (ab238262; Abcam).

### Bioinformatics

To conduct bioinformatic analysis, gene expression profiles were sourced from the Gene Expression Omnibus (GEO, http://www.ncbi.nlm.nih.gov/geo/) [24], selecting datasets GSE134431 [25], GSE143735 [26], and GSE199939. These datasets were downloaded, typically in.CEL or.txt format, and prepared for analysis. The next step involved pooling the data from these datasets, with an initial focus on normalizing and removing batch effects to ensure comparability. This was done using the “NormalizeBetweenArrays” and “RemoveBatchEffect” functions in the Limma R package, a widely-used tool for analyzing gene expression data. Principal Component Analysis (PCA) was then performed to understand the variance in the data, utilizing the “factoextra” R package for visualization. Finally, pathway enrichment analyses were conducted on genes that showed significant differences (p < 0.05) and at least a two-fold change in expression. This analysis was carried out using Metascape (http://metascape.org), an online tool that provides a detailed understanding of pathway involvement and interactions among the differentially expressed genes.

### Statistical analysis

For comparing multiple groups and two subgroups in our study, we employed a one-way analysis of variance (ANOVA) complemented by the Tukey post hoc method (GraphPad Software, version 9, La Jolla, CA, USA). A p-value of less than 0.05 was considered statistically significant, while non-significant differences were indicated as NS (p ≥ 0.05).

## Results

### Combination of public databases for analyzing altered genes in diabetic skin

**Fig. 1:**
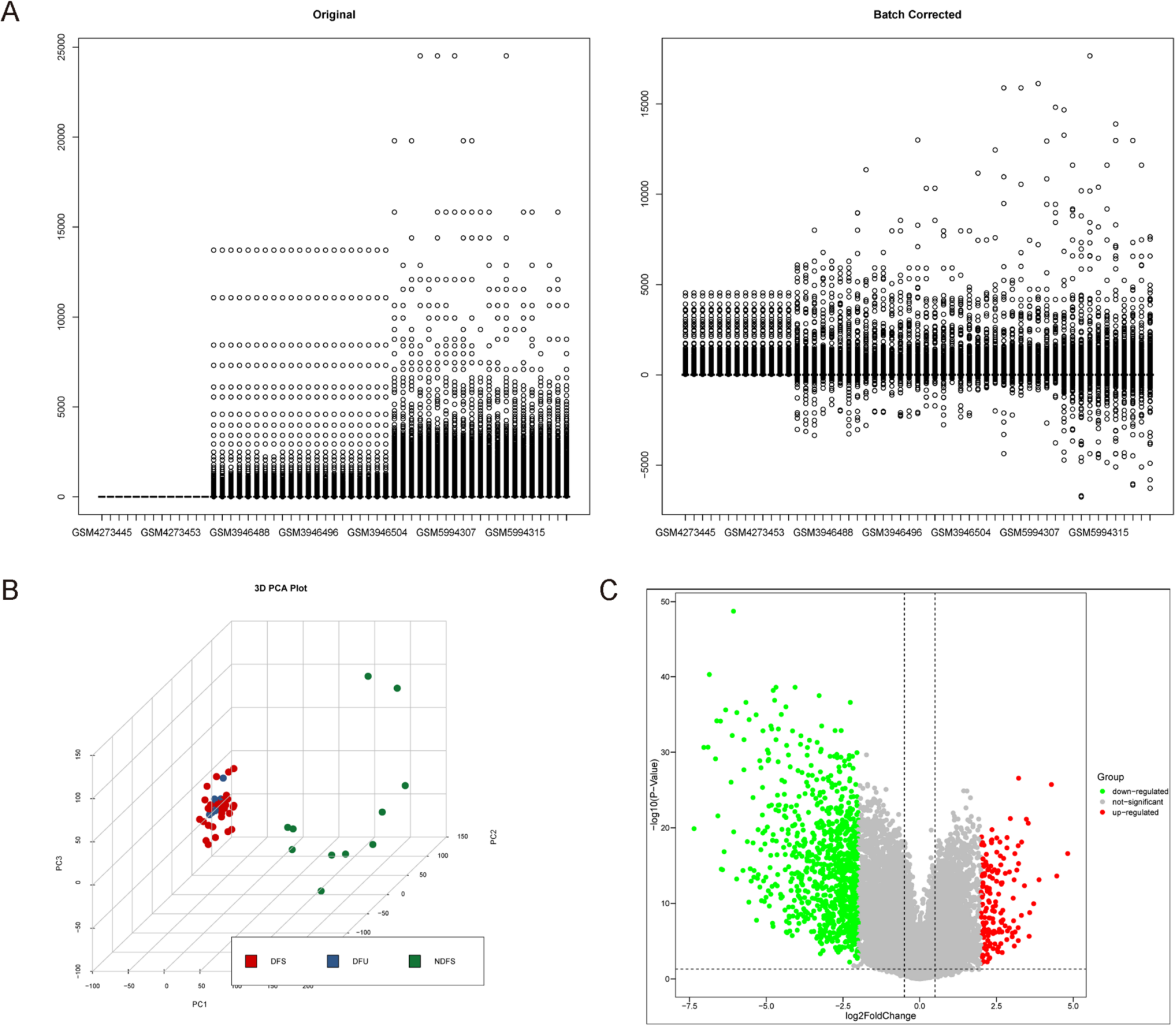
Combination of public databases for analyzing altered genes in diabetic skin. To increase the representativeness and the sample number in this analysis, we tried to include several public databases that allow the data to be pooled together. **A** Three gene expression profiles (GSE134431, GSE143735 and GSE199939) were specifically selected to remove the batch-effect, showing as boxplots before or after batch-effect removal. **B** Three types of the samples in the database were diabetic foot ulcer (DFU), diabetic foot skin (DFS) and non-diabetic foot skin (NDFS). PCA was then performed to demonstrate the distribution of the samples from different studies in a 3D mode. (C) A Volcano map to illustrate differentiated genes

First, we used bioinformatic tools to analyze selected public databases on normal and diabetic skin in patients. We included several databases that allow the data to be pooled together for analysis. After a careful assessment for relatedness and appropriateness, 3 gene expression profiles (GSE134431 [25], GSE143735 [26] and GSE199939) were specifically selected for analysis after removal of the batch-effect (Fig. 1A). The 3 types of the samples in the gene profiles were diabetic foot ulcer (DFU), diabetic foot skin (DFS) and non-diabetic foot skin (NDFS). PCA was then performed to demonstrate the distribution of the samples from different studies (Fig. 1B). We specifically compared the DFS with the NDFS, for which the significantly altered genes were shown in a Volcano map (Fig. 1C).

### Bioinformatic evidence for defective proinflammation and angiogenesis in diabetic skin

**Fig. 2:**
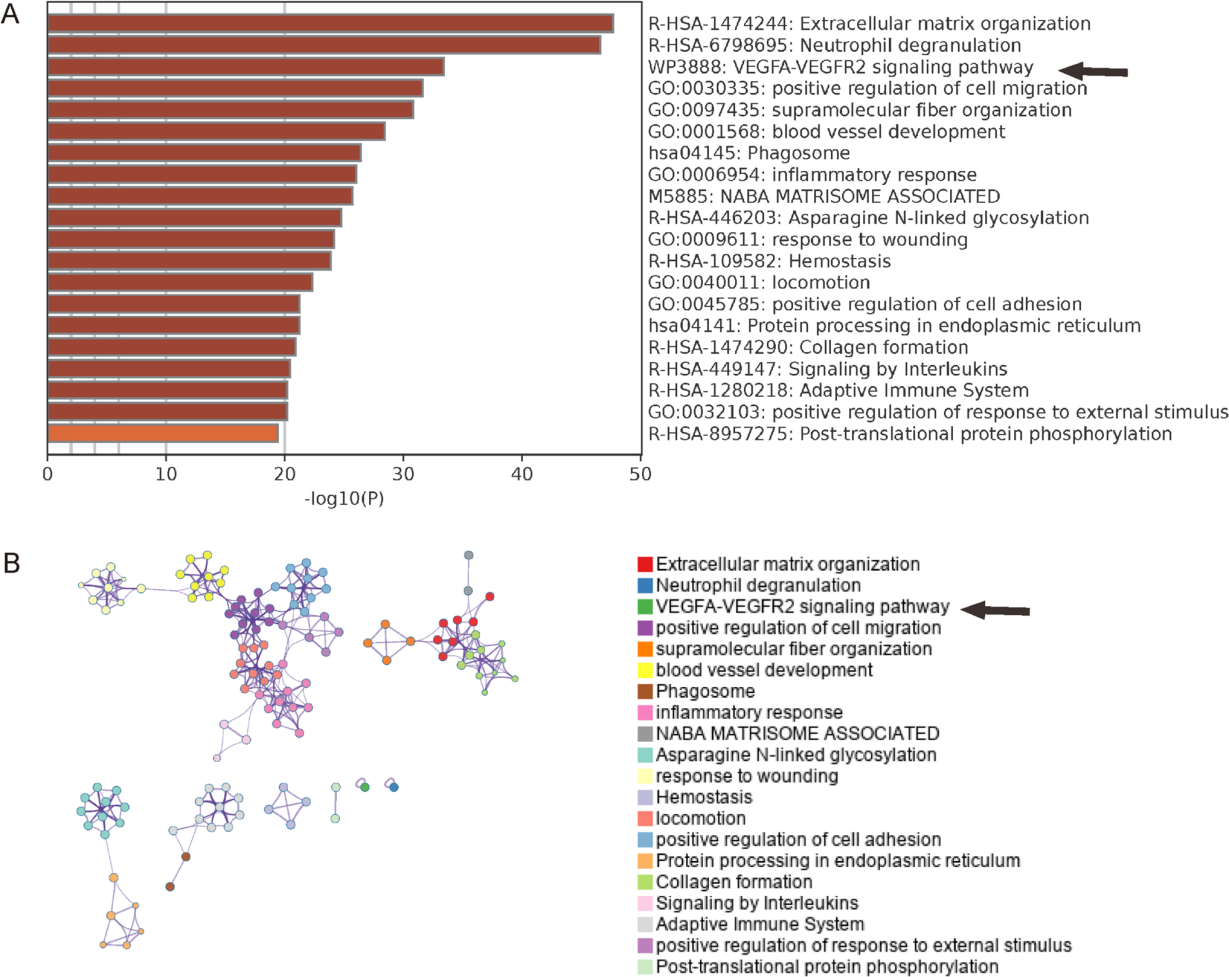
Bioinformatic evidence for defective proinflammation and angiogenesis in diabetic skin. **A**–**B** The significantly differed genes (p < 0.05) with at least a two-fold changes in values were subjected to a pathway enrichment analysis using the online tool Metascape (http://metascape.org), showing the top 20 greatest altered signal pathways in diabetes in a bar illustration (**A**) and in a network illustration (**B**). Arrows point to highlighted VEGF-VEGFR2 signaling pathway

The significantly differed genes (p < 0.05) with at least a two-fold changes in values were subjected to a pathway enrichment analysis using the online tool Metascape. The greatest altered signal pathways in diabetes were those associated with proinflammation and angiogenesis, such as extracellular matrix organization, neutrophil degranulation, VEGFA-VEGFR2 signaling pathway, positive regulation of cell migration, blood vessel development, phagosome, inflammatory response (Fig. 2A–B).

### Significant alterations in several key genes associated with proinflammation and angiogenesis in diabetic skin

**Fig. 3:**
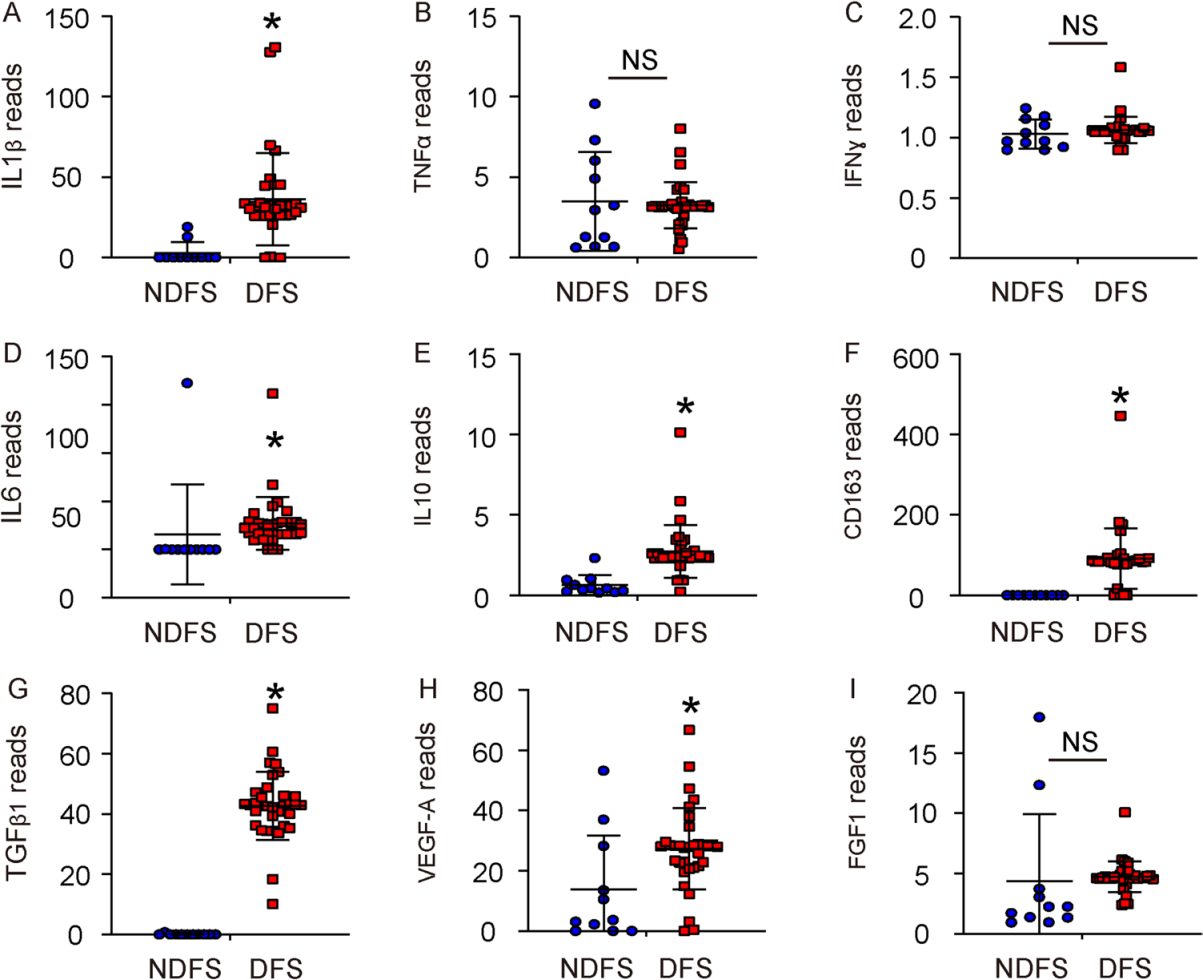
Significant alterations in several key genes associated with proinflammation and angiogenesis in diabetic skin. Levels of genes that are key regulators for proinflammation and angiogenesis were shown. **A** IL1β. **B** TNFα. **C** IFNɣ. **D** IL6. **E** IL10. **F** CD163. **G** TGFβ1. **H** VEGF-A. **I** FGF1. *p < 0.05. *NS* non-significant. *DFS* diabetic foot skin. *NDFS* non-diabetic foot skin

Next, we examined levels of the genes that are key regulators for proinflammation and angiogenesis. We found that IL1β levels were significantly increased in DFS, compared to NDFS (Fig. 3A). The TNFα and IFNɣ levels were not significantly altered (Fig. 3B–C). Moreover, IL6 and IL10 levels were significantly increased in DFS, compared to NDFS (Fig. 3D–E). These factors are associated with proinflammation, while IL10 also plays a role in macrophage polarization [27]. Thus, a proinflammatory status of diabetic skin may result from the activated status of IL1β, IL6 and IL10, which could cause a defective response during wound healing. We also detected a significantly increase in CD163 (Fig. 3F), an anti-inflammatory/M2 macrophage marker, a significantly increase in TGFβ1 (Fig. 3G), a factor associated with macrophage polarization and fibroblast activation, and a significantly increase in VEGF-A (Fig. 3H), a potent angiogenetic factor in DFS, compared to NDFS, although we did not detect significant alteration in FGF1 (Fig. 3I). These data suggest that macrophages in diabetic skin may be polarized to M2-like at quiescence, while the fibrosis and angiogenesis pathway in diabetic skin were both pre-activated before a wound.

### Defect in wound proinflammatory/M1 macrophages and fibroblasts in diabetic mice

**Fig. 4:**
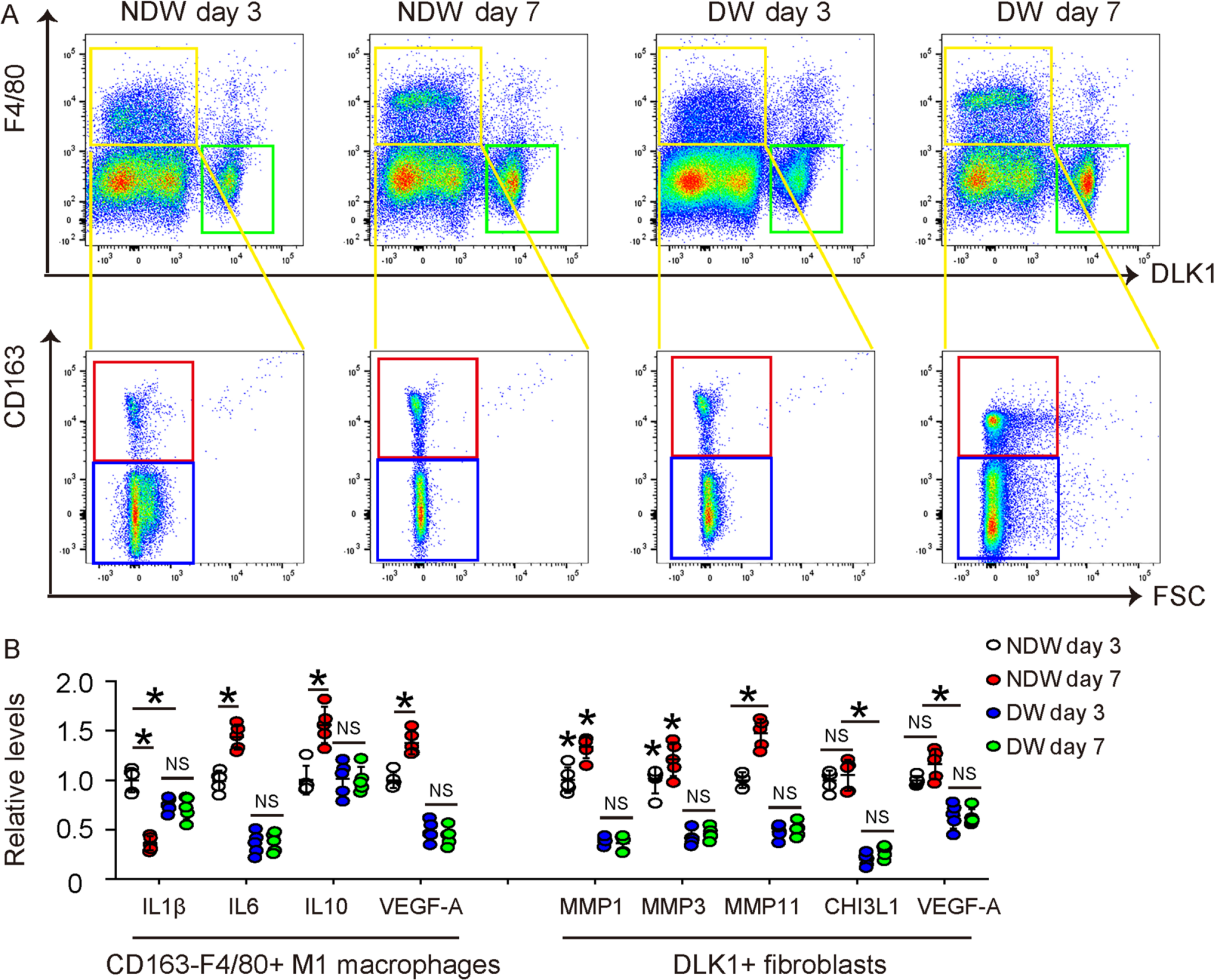
Defect in wound proinflammatory/M1 macrophages and fibroblasts in diabetic mice. **A** In a mouse model that creates a skin wound in STZ-treated diabetic mice, fibroblasts (DLK1 +), M1 macrophages (F4/80 + CD163-) and M2 macrophages (F4/80 + CD163 +) were isolated from the wound tissue by flow cytometry, shown by representative flow charts. **B** ELISA for IL1β, IL6, IL10 and VEGF-A in M1 macrophages and for MMP1, MMP3, MMP11, CHI3L1 and VEGF-A in fibroblasts. *p < 0.05. *NS* non-significant. *NDW* non-diabetic wound. *DW* Diabetic wound

We hypothesized the pre-activated status of proinflammation and angiogenesis in diabetic skin may impair their proper activation when a wound occurs. Since no human specimens allow such studies, we used a mouse model for diabetic wound healing to address this question. This model has been described in our previous studies [23, 28, 29]. It is known that proinflammatory/M1 macrophages dominate in the inflammatory phase of wound healing and their number peaks at day 3 after the wound, while anti-inflammatory/M2 macrophage dominate in the proliferative phase and their number peaks at day 7 after the wound [6]. Thus, we isolated fibroblasts (DLK1 +), M1 macrophages (F4/80 + CD163-) and M2 macrophages (F4/80 + CD163 +) from the mouse wound tissue by flow cytometry (Fig. 4A). We examined levels of key factors that are associated with proinflammation and significantly altered in diabetes (IL1β, IL6 and IL10; Fig. 3A, D, E) and that are associated with angiogenesis (VEGF-A, Fig. 3H) in M1 macrophages (Fig. 4B). We also examined levels of marker factors in the healing-associated fibroblasts (MMP1, MMP3, MMP11, CHI3L1 and VEGF-A) in fibroblasts by ELISA (Fig. 4B). Interestingly, the significant alteration in levels of IL1β, IL6, IL10 and VEGF-A in non-diabetic M1 macrophages between day 3 and day 7 after the wound were all abolished in diabetic M1 macrophages (Fig. 4B). Moreover, the significant alteration in levels of MMP1, MMP3, MMP11, CHI3L1 and VEGF-A in non-diabetic fibroblasts between day 3 and day 7 after the wound were all abolished in diabetic M1 fibroblasts, and their expression levels were also significantly lower in diabetes (Fig. 4B). These data suggest that the diabetic status may cause the loss of the spatiotemporal response of both M1 macrophages and fibroblasts to the wound, in terms of their phenotypic adaptation and release of cytokines as a possible reason for the defective wound healing in diabetes.

### M1/proinflammatory macrophages regulate the phenotype of fibroblasts

**Fig. 5:**
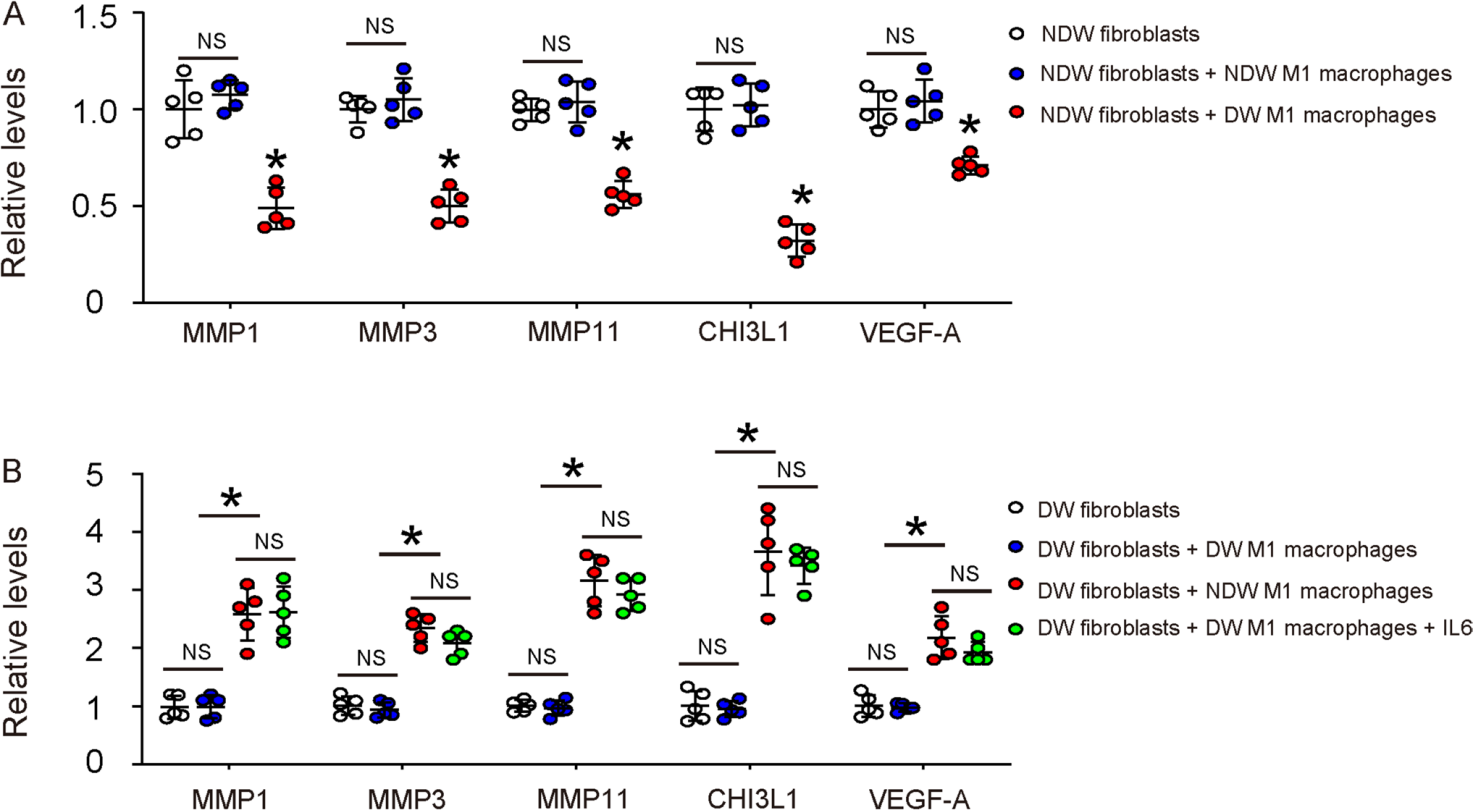
M1/proinflammatory macrophages regulate the phenotype of fibroblasts. **A** Fibroblasts isolated from non-diabetic wound were cultured alone, or with isolated M1 macrophages from non-diabetic wound, or with isolated M1 macrophages from diabetic wound. ELISA for MMP1, MMP3, MMP11, CHI3L1 and VEGF-A in fibroblasts was performed. **B** Fibroblasts isolated from diabetic wound were cultured alone, or with isolated M1 macrophages from diabetic wound supplied with/without IL6, or with isolated M1 macrophages from non-diabetic wound. ELISA for MMP1, MMP3, MMP11, CHI3L1 and VEGF-A in fibroblasts was performed. *p < 0.05. *NS* non-significant. *NDW* non-diabetic wound. *DW* Diabetic wound

In order to find out whether M1/proinflammatory macrophages regulate the phenotypic change and activation of healing-associated fibroblasts in wound, we did 2 co-culture experiments. First, fibroblasts isolated from non-diabetic wound were cultured alone, or with isolated M1 macrophages from non-diabetic wound, or with isolated M1 macrophages from diabetic wound. The levels of those key factors for healing were examined in fibroblasts. We found that co-culture with isolated M1 macrophages from non-diabetic wound did not significantly alter the levels of MMP1, MMP3, MMP11, CHI3L1 and VEGF-A in fibroblasts from non-diabetic wound (Fig. 5A). However, co-culture with isolated M1 macrophages from diabetic wound significantly decreased the levels of MMP1, MMP3, MMP11, CHI3L1 and VEGF-A in fibroblasts from non-diabetic wound (Fig. 5A). In the second experiment, fibroblasts isolated from diabetic wound were cultured alone, or with isolated M1 macrophages from diabetic wound, or with isolated M1 macrophages from non-diabetic wound. The levels of those key factors for healing in fibroblasts were examined. We found that co-culture with isolated M1 macrophages from diabetic wound did not significantly alter the levels of MMP1, MMP3, MMP11, CHI3L1 and VEGF-A in fibroblasts from diabetic wound (Fig. 5B). However, co-culture with isolated M1 macrophages from non-diabetic wound significantly increased the levels of MMP1, MMP3, MMP11, CHI3L1 and VEGF-A in fibroblasts from diabetic wound (Fig. 5B). Moreover, adding IL6 into the co-culture of fibroblasts isolated from diabetic wound with isolated M1 macrophages from diabetic wound mimicked the effects of co-culture of fibroblasts isolated from diabetic wound with isolated M1 macrophages from non-diabetic wound (Fig. 5B). Together, these data suggest that M1/proinflammatory macrophages regulate the phenotype of fibroblasts, and the defect in diabetic M1 macrophages could be corrected by IL6, at least for their effects on fibroblast activation.

### Generation of AAVs that specifically express IL6 in macrophages

**Fig. 6:**
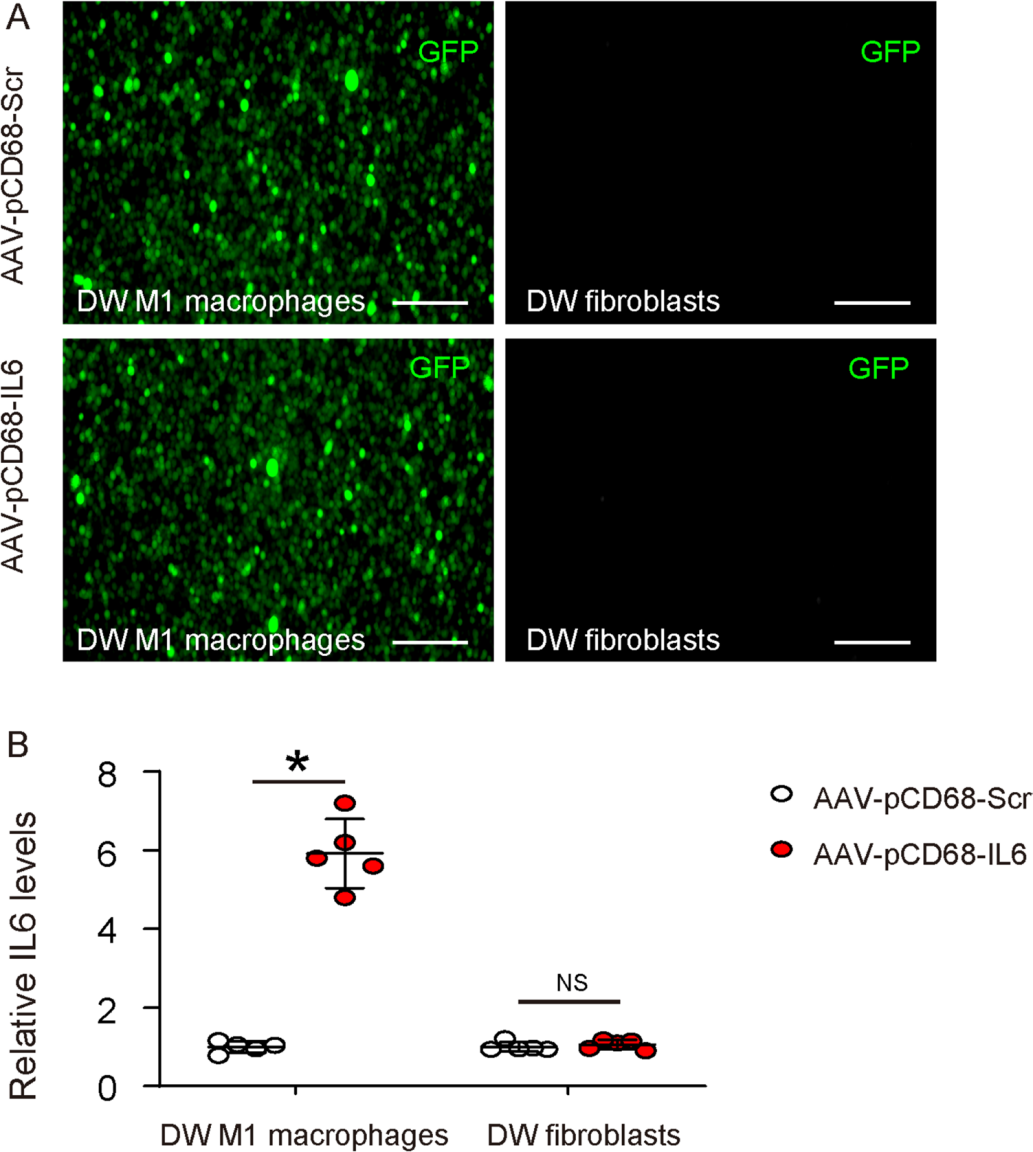
Generation of AAVs that express IL6 specifically in macrophages. We generated an AAV carrying IL6 under a macrophage-specific CD68 promoter (AAV-pCD68-IL6), which allows specific expression of IL6 in skin macrophages after a local administration for assessing its effect on diabetic wound healing. **A** To ensure the specificity of CD68 promoter for macrophage and specifically not for fibroblasts, isolated M1 macrophages and fibroblasts from diabetic wound were infected with either AAV-pCD68-IL6 or control AAV-pCD68-Scr. GFP was used to detect infection. **B** ELISA for IL6 in infected cells. *p < 0.05. NS: non-significant. Scale bars are 100 µm. *DW* Diabetic wound

In order to extrapolate our in vitro finding to in vivo, we generated an AAV carrying IL6 under a macrophage-specific CD68 promoter (AAV-pCD68-IL6), which allows specific expression of IL6 in skin macrophages after a local administration for assessing its effect on diabetic wound healing (Fig. 3A). To ensure the specificity of CD68 promoter for macrophage and specifically not for fibroblasts, either AAV-pCD68-IL6 or control AAV-pCD68-Scr were used to transduce isolated M1 macrophages and fibroblasts from diabetic wound. Our result based viral GFP expression showed specific transduction of macrophages, but not transduction of fibroblasts, by these viruses (Fig. 6A). The levels of IL6 were then determined, which further proved the specificity of the CD68 promoter for macrophages (Fig. 6B).

### Macrophage-expression of IL6 does not affect diabetes

**Fig. 7:**
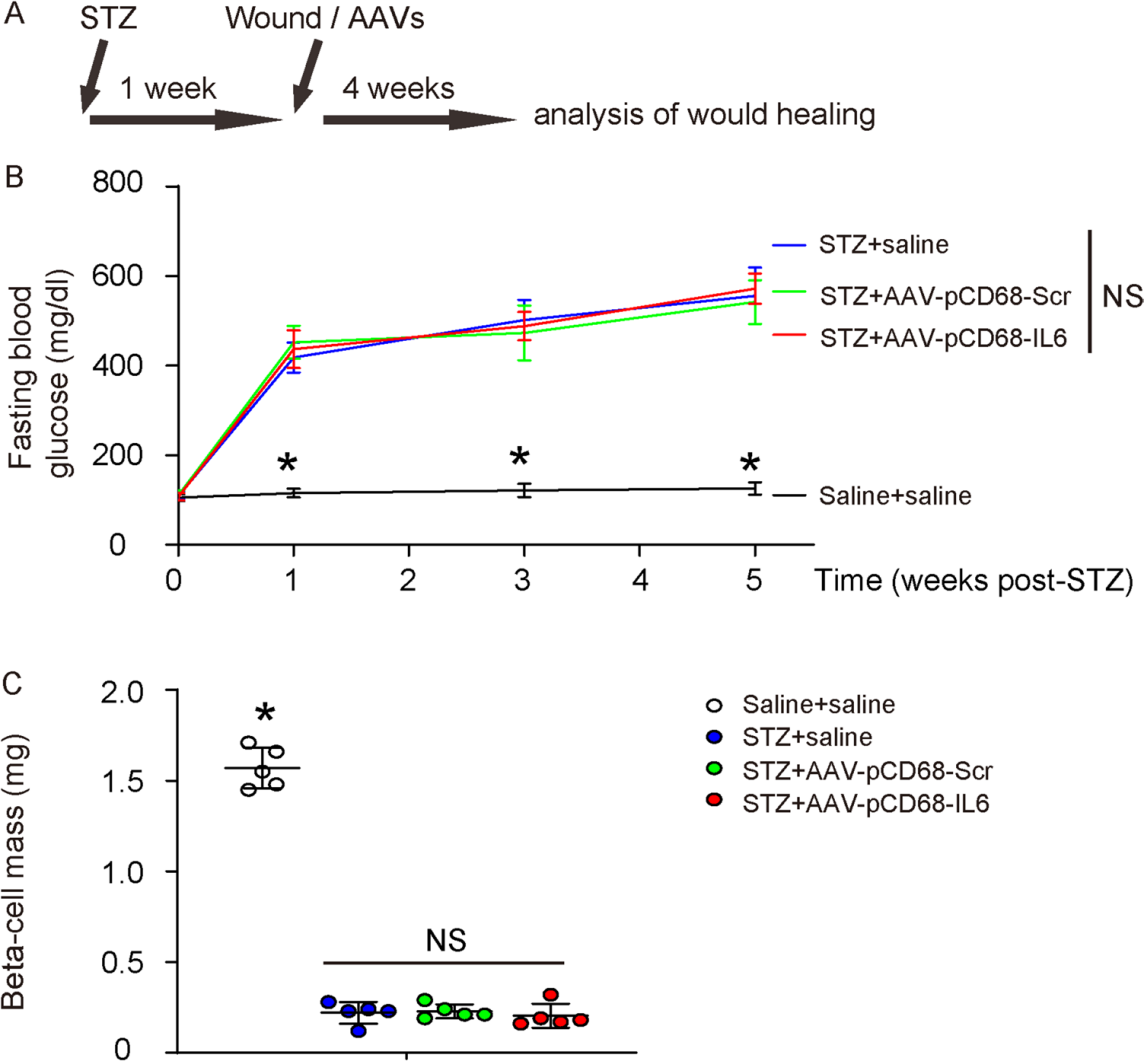
Macrophage-expression of IL6 does not affect diabetes. **A** Schematic of a mouse model for diabetic wound healing, in which STZ-tread diabetic mice received a surgical creation of a skin wound as well as an immediate local viral injection. Mice were kept for another 4 weeks for analysis. Four groups of mice of 5 each were applied in the experiment. Group 1: mice received i.p. saline and local saline (saline + salilne). Group 2: mice received i.p. STZ and local saline (STZ + saline). Group 3: mice received i.p. STZ and local control virus (STZ + AAV-pCD68-Scr); Group 4: mice received i.p. STZ and local IL6 virus (STZ + AAV-pCD68-IL6). **B** Fasting blood glucose. **C** Beta-cell mass at sacrifice. *p < 0.05. *NS* non-significant

The effects of macrophage-IL6 on diabetic wound healing were then examined in the described mouse model, in which STZ-tread diabetic mice received a surgical creation of a skin wound as well as a simultaneous local viral injection [23]. After another 4 weeks were used to monitor the wound healing of the mice (Fig. 7A). Four groups of mice of 5 each were applied in the experiment. Group 1: mice received i.p. saline and local saline (saline + saline). Group 2: mice received i.p. STZ and local saline (STZ + saline). Group 3: mice received i.p. STZ and local control virus (STZ + AAV-pCD68-Scr); Group 4: mice received i.p. STZ and local IL6 virus (STZ + AAV-pCD68-IL6). Neither the induction of an irreversible hyperglycemia (Fig. 7B) nor the value of beta-cell mass (Fig. 7C) was altered by viral injection, suggesting that the viral treatments here do not affect diabetes.

### Macrophage-expression of IL6 promotes wound healing and angiogenesis

**Fig. 8:**
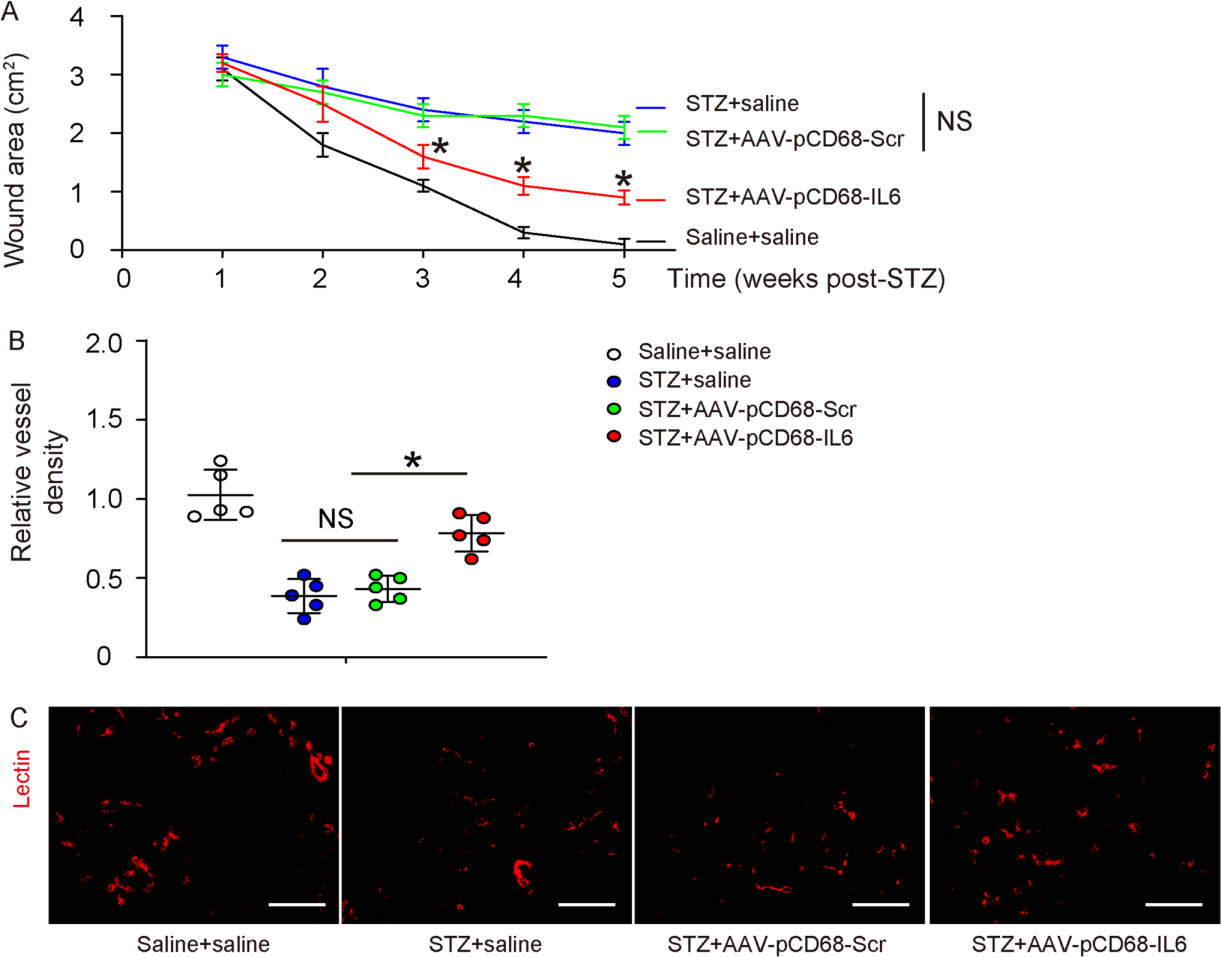
Macrophage-expression of IL6 promotes wound healing and angiogenesis. **A** Wound area measurement. **B**–**C** Angiogenesis was analyzed at the end of the experiment by vessel density based on relative lectin area, shown by quantification (**B**) and by representative fluorescent images (**C**). *p < 0.05. NS: non-significant. Scale bars are 100 µm

Next, we examined the effects of macrophage-expression of IL6 on diabetic wound healing. We found that the wound was cured by more than 95% at 4 weeks in saline-treated non-diabetic mice (i.p control for STZ and local injection control for virus) (Fig. 8A). The wound was cured for less than 30% in STZ-treated mice with local injection of saline or control viral (Fig. 8A). However, the wound was cured for about 65% in STZ-treated mice with local IL6 viral injection, which was significantly adventurous over the other two STZ-treated groups (Fig. 8A). This improvement of wound recovery by IL6 expression in macrophages may be partially due to the improved angiogenesis, which was quantified by lectin-perfused area in the wound (Fig. 8B–C). Together, our in vitro and in vivo data suggest that M1/proinflammatory macrophages may regulate the induction of healing-associated fibroblasts.

## Discussion

The phenotypic changes of proinflammatory/M1 macrophages in diabetes may result from two factors. First, an analysis of public database from patients showed that diabetic status could render a quiescent skin (no wound) to be proinflammatory. Thus, the continuous presence of inflammation in diabetes thus might be harmful to local microenvironment in the skin, causing a sustained and modest activation of macrophages, fibroblasts and keratinocytes, as well as reducing their responsive potential when a wound occurs [30]. Second, since all phases of a normal wound healing are impaired in diabetes, the interaction among macrophages, fibroblasts and other cell types could be further damaged [31]. For example, in the hemostasis phase, the vasoconstriction, platelet aggregation, clot formation and platelet degranulation to form insoluble fibrin for neutrophil recruitment and activation are all altered in a long hyperglycemic status [6, 32, 33]. Hence, the activation of macrophages by activated neutrophils is altered in diabetes [34]. In the inflammatory and proliferative phases, the crosstalk between macrophages and fibroblasts appears to be very important for the clearance of dead tissue, initiation of angiogenesis and progression of tissue repair [35, 36]. Specifically, macrophages orchestrate the complicated cell–cell crosstalk to fulfill all the biological activities required for a successful wound healing [37]. A spatiotemporal change in macrophages is very impressive and required for a wound healing to occur properly, as shown here and in previous studies [36, 38].

Here, we specifically detected reduced expressions of IL1β, IL6, IL10 and VEGF-A in M1/proinflammatory macrophages at the inflammatory stage in diabetes. IL1β is very critical for proinflammatory activation and activates different signaling pathways such as MAPK, NF-kappa B, PI3-K/Akt and MEK/ERK [39]. The compensated IL1β activation in diabetes perhaps caused incompleteness of adequate immune reaction [40]. IL10 is an anti-inflammatory cytokine with essential immunoregulatory functions [41]. Impaired IL10 activation could lead to incompleteness of inflammatory solution and failure of proper phenotypic transition of proinflammatory macrophages and other cell types [42]. IL6 a pleiotropic cytokine that plays a vital role in wound healing, while wound healing is damaged due to loss of IL6 itself [43]. Moreover, high IL6 levels have been shown to be advantageous for DFUs [44]. IL6 regulates many cell types including macrophages, lymphocytes and fibroblasts [16]. Therefore, we used a macrophage-IL6 to correct the impaired effects of proinflammatory/M1 macrophages on fibroblast activation in this study. VEGF-A is the well-known and most potent angiogenetic factor [45]. The impaired cure of diabetic wound is at least due to impaired angiogenesis [46]. Therefore, it is not surprising that VEGF-A from both macrophages and fibroblasts was reduced in diabetes, and the improvement of diabetic wound healing by macrophage-expression of IL6 also improved angiogenesis through VEGF-A.

The proposition that IL6 holds therapeutic potential in wound healing, particularly in diabetic contexts, is indeed significant [43]. IL6, traditionally classified as a pro-inflammatory cytokine, plays a critical role in immune responses and has been observed to aid in tissue repair and regeneration [43]. This dual nature presents a unique opportunity for its use in therapeutic interventions for diabetic wounds, which often suffer from impaired healing processes. However, the employment of IL6 as a treatment necessitates a thorough examination of its safety profile, especially given its pro-inflammatory properties [44]. In the context of diabetes, a condition often accompanied by chronic inflammation, there is a legitimate concern that exogenous IL6 could exacerbate underlying inflammatory states, potentially leading to worsened diabetic complications. For instance, elevated levels of IL6 have been associated with insulin resistance and the progression of diabetic complications like nephropathy and retinopathy [44]. Therefore, a detailed investigation into the possible side effects of IL6 therapy is crucial. This should encompass not only the immediate inflammatory responses but also long-term effects on metabolic control and diabetes-related complications. Moreover, determining the optimal dosage and delivery method that maximizes wound healing benefits while minimizing pro-inflammatory risks is essential. Balancing IL6's regenerative capabilities against its inflammatory actions will be key in developing a therapeutic strategy that is both effective and safe for patients with diabetes.

A very recent study reported that about 10% of wound fibroblasts were likely originated from monocytes/macrophages, suggesting a plasticity of macrophages during wound healing [30]. However, the contribution of macrophages to healing fibroblasts was never investigated either previously or in the current study. It would be interesting to apply a lineage tracing approach to address this question in the future.

Using mouse models in wound healing research, especially for studying human DFUs, is both valuable and challenging [3]. Their primary advantage lies in the ease of genetic manipulation, which allows for detailed studies of specific genes and molecular pathways involved in wound healing under diabetic conditions [3]. This can be crucial for uncovering novel therapeutic targets. Mice also offer a controlled environment, where factors like diet, genetics, and environmental conditions are standardized, leading to more consistent and replicable results [3]. However, the limitations are noteworthy. Mice significantly differ from humans in skin structure, immune responses, and wound healing mechanisms, which can affect the translatability of findings to human DFUs [3]. Additionally, the diabetic state in mice is often induced artificially, which might not fully replicate the complexity of human diabetes and its impact on wound healing [3]. Thus, while mouse models are invaluable for preliminary investigations and understanding basic mechanisms, the applicability of these findings to human DFUs must be approached with caution, often necessitating further validation in human tissues or clinical trials.

## Conclusions

M1/proinflammatory macrophages play a critical role in activating fibroblasts associated with wound healing. However, in diabetes, these M1/proinflammatory macrophages often exhibit a compromised ability to stimulate fibroblast activation, impeding the healing process. The potential of IL6 treatment emerges as a promising therapeutic strategy to address this deficiency. By enhancing the function of defective M1/proinflammatory macrophages, IL6 could effectively restore their capacity to activate wound-healing fibroblasts, thereby improving the treatment outcomes for DFUs.

## Data Availability

The datasets generated during and/or analyzed during the current study are available from the corresponding author on reasonable request.

## References

[1] Bluestone JA, Herold K, Eisenbarth G (2010) Genetics, pathogenesis and clinical interventions in type 1 diabetes. Nature 464(7293):1293–1300 20432533 10.1038/nature08933 PMC4959889

[2] Ali MK, Pearson-Stuttard J, Selvin E, Gregg EW (2022) Interpreting global trends in type 2 diabetes complications and mortality. Diabetologia 65(1):3–13 34837505 10.1007/s00125-021-05585-2 PMC8660730

[3] American Diabetes A (2021) 11 Microvascular complications and foot care: standards of medical care in diabetes-2021. Diabetes Care 44(Suppl 1):S151–S167 33298422 10.2337/dc21-S011

[4] Manickum P, Mashamba-Thompson T, Naidoo R, Ramklass S, Madiba T (2021) Knowledge and practice of diabetic foot care—a scoping review. Diabetes Metab Syndr 15(3):783–793 33838615 10.1016/j.dsx.2021.03.030

[5] Wolf SJ, Melvin WJ, Gallagher K (2021) Macrophage-mediated inflammation in diabetic wound repair. Semin Cell Dev Biol 119:111–118 34183242 10.1016/j.semcdb.2021.06.013 PMC8985699

[6] Louiselle AE, Niemiec SM, Zgheib C, Liechty KW (2021) Macrophage polarization and diabetic wound healing. Transl Res 236:109–116 34089902 10.1016/j.trsl.2021.05.006

[7] Liu BF, Miyata S, Kojima H, Uriuhara A, Kusunoki H, Suzuki K, Kasuga M (1999) Low phagocytic activity of resident peritoneal macrophages in diabetic mice: relevance to the formation of advanced glycation end products. Diabetes 48(10):2074–2082 10512376 10.2337/diabetes.48.10.2074

[8] Rungratanawanich W, Qu Y, Wang X, Essa MM, Song BJ (2021) Advanced glycation end products (AGEs) and other adducts in aging-related diseases and alcohol-mediated tissue injury. Exp Mol Med 53(2):168–188 33568752 10.1038/s12276-021-00561-7 PMC8080618

[9] Bannon P, Wood S, Restivo T, Campbell L, Hardman MJ, Mace KA (2013) Diabetes induces stable intrinsic changes to myeloid cells that contribute to chronic inflammation during wound healing in mice. Dis Model Mech 6(6):1434–1447 24057002 10.1242/dmm.012237 PMC3820266

[10] Miao M, Niu Y, Xie T, Yuan B, Qing C, Lu S (2012) Diabetes-impaired wound healing and altered macrophage activation: a possible pathophysiologic correlation. Wound Repair Regen 20(2):203–213 22380690 10.1111/j.1524-475X.2012.00772.x

[11] Khanna S, Biswas S, Shang Y, Collard E, Azad A, Kauh C, Bhasker V, Gordillo GM, Sen CK, Roy S (2010) Macrophage dysfunction impairs resolution of inflammation in the wounds of diabetic mice. PLoS ONE 5(3):e9539 20209061 10.1371/journal.pone.0009539 PMC2832020

[12] Mirza RE, Fang MM, Ennis WJ, Koh TJ (2013) Blocking interleukin-1beta induces a healing-associated wound macrophage phenotype and improves healing in type 2 diabetes. Diabetes 62(7):2579–2587 23493576 10.2337/db12-1450 PMC3712034

[13] Mirza R, Koh TJ (2011) Dysregulation of monocyte/macrophage phenotype in wounds of diabetic mice. Cytokine 56(2):256–264 21803601 10.1016/j.cyto.2011.06.016

[14] Munadziroh E, Putri GA, Ristiana V, Agustantina TH, Nirwana I, Razak FA, Surboyo MDC (2022) The role of recombinant secretory leukocyte protease inhibitor to CD163, FGF-2, IL-1 and IL-6 expression in skin wound healing. Clin Cosmet Investig Dermatol 15:903–910 35611048 10.2147/CCID.S358897 PMC9124476

[15] Liu C, Xu Y, Lu Y, Du P, Li X, Wang C, Guo P, Diao L, Lu G (2022) Mesenchymal stromal cells pretreated with proinflammatory cytokines enhance skin wound healing via IL-6-dependent M2 polarization. Stem Cell Res Ther 13(1):414 35964139 10.1186/s13287-022-02934-9 PMC9375394

[16] Johnson BZ, Stevenson AW, Prele CM, Fear MW, Wood FM (2020) The role of IL-6 in skin fibrosis and cutaneous wound healing. Biomedicines 8(5):101 32365896 10.3390/biomedicines8050101 PMC7277690

[17] Theocharidis G, Thomas BE, Sarkar D, Mumme HL, Pilcher WJR, Dwivedi B, Sandoval-Schaefer T, Sirbulescu RF, Kafanas A, Mezghani I, Wang P, Lobao A, Vlachos IS et al (2022) Single cell transcriptomic landscape of diabetic foot ulcers. Nat Commun 13(1):181 35013299 10.1038/s41467-021-27801-8 PMC8748704

[18] Yanagisawa H, Yokoyama U (2021) Extracellular matrix-mediated remodeling and mechanotransduction in large vessels during development and disease. Cell Signal 86:110104 34339854 10.1016/j.cellsig.2021.110104

[19] Ahmad K, Choi I, Lee YH (2020) Implications of skeletal muscle extracellular matrix remodeling in metabolic disorders: diabetes perspective. Int J Mol Sci 21(11):3845 32481704 10.3390/ijms21113845 PMC7312063

[20] Gray JT, Zolotukhin S (2011) Design and construction of functional AAV vectors. Methods Mol Biol 807:25–46 22034025 10.1007/978-1-61779-370-7_2

[21] Lang R, Rutschman RL, Greaves DR, Murray PJ (2002) Autocrine deactivation of macrophages in transgenic mice constitutively overexpressing IL-10 under control of the human CD68 promoter. J Immunol 168(7):3402–3411 11907098 10.4049/jimmunol.168.7.3402

[22] Manderson AP, Kay JG, Hammond LA, Brown DL, Stow JL (2007) Subcompartments of the macrophage recycling endosome direct the differential secretion of IL-6 and TNFalpha. J Cell Biol 178(1):57–69 17606866 10.1083/jcb.200612131 PMC2064421

[23] Zhu L, Qian J, Jiang Y, Yang T, Duan Q, Xiao X (2021) PlGF reduction compromises angiogenesis in diabetic foot disease through macrophages. Front Immunol 12:736153 34659227 10.3389/fimmu.2021.736153 PMC8511710

[24] Barrett T, Wilhite SE, Ledoux P, Evangelista C, Kim IF, Tomashevsky M, Marshall KA, Phillippy KH, Sherman PM, Holko M, Yefanov A, Lee H, Zhang N et al (2013) NCBI GEO: archive for functional genomics data sets–update. Nucleic Acids Res 41:D991-995 23193258 10.1093/nar/gks1193 PMC3531084

[25] Sawaya AP, Stone RC, Brooks SR, Pastar I, Jozic I, Hasneen K, O’Neill K, Mehdizadeh S, Head CR, Strbo N, Morasso MI, Tomic-Canic M (2020) Deregulated immune cell recruitment orchestrated by FOXM1 impairs human diabetic wound healing. Nat Commun 11(1):4678 32938916 10.1038/s41467-020-18276-0 PMC7495445

[26] Theocharidis G, Baltzis D, Roustit M, Tellechea A, Dangwal S, Khetani RS, Shu B, Zhao W, Fu J, Bhasin S, Kafanas A, Hui D, Sui SH et al (2020) Integrated skin transcriptomics and serum multiplex assays reveal novel mechanisms of wound healing in diabetic foot ulcers. Diabetes 69(10):2157–2169 32763913 10.2337/db20-0188 PMC7506837

[27] Mantovani A, Dinarello CA, Molgora M, Garlanda C (2019) Interleukin-1 and related cytokines in the regulation of inflammation and immunity. Immunity 50(4):778–795 30995499 10.1016/j.immuni.2019.03.012 PMC7174020

[28] Zhu L, Wang G, Fischbach S, Xiao X (2017) Suppression of microRNA-205-5p in human mesenchymal stem cells improves their therapeutic potential in treating diabetic foot disease. Oncotarget 8(32):52294–52303 28881730 10.18632/oncotarget.17012 PMC5581029

[29] Zhu L, Zhong Q, Yang T, Xiao X (2019) Improved therapeutic effects on diabetic foot by human mesenchymal stem cells expressing MALAT1 as a sponge for microRNA-205-5p. Aging (Albany NY) 11(24):12236–12245 31866580 10.18632/aging.102562 PMC6949052

[30] Guerrero-Juarez CF, Dedhia PH, Jin S, Ruiz-Vega R, Ma D, Liu Y, Yamaga K, Shestova O, Gay DL, Yang Z, Kessenbrock K, Nie Q, Pear WS et al (2019) Single-cell analysis reveals fibroblast heterogeneity and myeloid-derived adipocyte progenitors in murine skin wounds. Nat Commun 10(1):650 30737373 10.1038/s41467-018-08247-x PMC6368572

[31] Matoori S, Veves A, Mooney DJ (2021) Advanced bandages for diabetic wound healing. Sci Transl Med. 10.1126/scitranslmed.abe4839 33731435 10.1126/scitranslmed.abe4839

[32] Mirza R, DiPietro LA, Koh TJ (2009) Selective and specific macrophage ablation is detrimental to wound healing in mice. Am J Pathol 175(6):2454–2462 19850888 10.2353/ajpath.2009.090248 PMC2789630

[33] Clark RA (2003) Fibrin is a many splendored thing. J Invest Dermatol. 121(5):xxi–xxii 14708590 10.1046/j.1523-1747.2003.12575.x

[34] Mu X, Wu X, He W, Liu Y, Wu F, Nie X (2022) Pyroptosis and inflammasomes in diabetic wound healing. Front Endocrinol (Lausanne) 13:950798 35992142 10.3389/fendo.2022.950798 PMC9389066

[35] Minutti CM, Knipper JA, Allen JE, Zaiss DM (2017) Tissue-specific contribution of macrophages to wound healing. Semin Cell Dev Biol 61:3–11 27521521 10.1016/j.semcdb.2016.08.006

[36] Sharifiaghdam M, Shaabani E, Faridi-Majidi R, De Smedt SC, Braeckmans K, Fraire JC (2022) Macrophages as a therapeutic target to promote diabetic wound healing. Mol Ther 30(9):2891–2908 35918892 10.1016/j.ymthe.2022.07.016 PMC9482022

[37] Hesketh M, Sahin KB, West ZE, Murray RZ (2017) Macrophage phenotypes regulate scar formation and chronic wound healing. Int J Mol Sci 18(7):1545 28714933 10.3390/ijms18071545 PMC5536033

[38] Rahmannia M, Amini A, Chien S, Bayat M (2022) Impact of photobiomodulation on macrophages and their polarization during diabetic wound healing: a systematic review. Lasers Med Sci 37(7):2805–2815 35635648 10.1007/s10103-022-03581-5

[39] Weber A, Wasiliew P, Kracht M (2010) Interleukin-1beta (IL-1beta) processing pathway. Sci Signal 3(105):cm2 20086236 10.1126/scisignal.3105cm2

[40] Chen X, Zhang D, Li Y, Wang W, Bei W, Guo J (2021) NLRP3 inflammasome and IL-1beta pathway in type 2 diabetes and atherosclerosis: friend or foe? Pharmacol Res 173:105885 34536551 10.1016/j.phrs.2021.105885

[41] Dimitrijevic M, Stanojevic S, Vujic V, Aleksic I, Pilipovic I, Leposavic G (2014) Aging oppositely affects TNF-alpha and IL-10 production by macrophages from different rat strains. Biogerontology 15(5):475–486 25009084 10.1007/s10522-014-9513-4

[42] Abdoli A, Maspi N, Ghaffarifar F (2017) Wound healing in cutaneous leishmaniasis: a double edged sword of IL-10 and TGF-beta. Comp Immunol Microbiol Infect Dis 51:15–26 28504090 10.1016/j.cimid.2017.02.001

[43] Lin ZQ, Kondo T, Ishida Y, Takayasu T, Mukaida N (2003) Essential involvement of IL-6 in the skin wound-healing process as evidenced by delayed wound healing in IL-6-deficient mice. J Leukoc Biol 73(6):713–721 12773503 10.1189/jlb.0802397

[44] Pradhan L, Cai X, Wu S, Andersen ND, Martin M, Malek J, Guthrie P, Veves A, Logerfo FW (2011) Gene expression of pro-inflammatory cytokines and neuropeptides in diabetic wound healing. J Surg Res 167(2):336–342 20070982 10.1016/j.jss.2009.09.012 PMC4376536

[45] Xiao X, Guo P, Chen Z, El-Gohary Y, Wiersch J, Gaffar I, Prasadan K, Shiota C, Gittes GK (2013) Hypoglycemia reduces vascular endothelial growth factor a production by pancreatic beta cells as a regulator of Beta cell mass. J Biol Chem 288(12):8636–8646 23378532 10.1074/jbc.M112.422949 PMC3605682

[46] Han C, Barakat M, DiPietro LA (2022) Angiogenesis in wound repair: too much of a good thing? Cold Spring Harb Perspect Biol 14(10):a041225 35667793 10.1101/cshperspect.a041225 PMC9524283

